# Discovery of biological networks from diverse functional genomic data

**DOI:** 10.1186/gb-2005-6-13-r114

**Published:** 2005-12-19

**Authors:** Chad L Myers, Drew Robson, Adam Wible, Matthew A Hibbs, Camelia Chiriac, Chandra L Theesfeld, Kara Dolinski, Olga G Troyanskaya

**Affiliations:** 1 Department of Computer Science, Princeton University, 35 Olden Street, Princeton, NJ 08544, USA; 2 Lewis-Sigler Institute for Integrative Genomics, Carl Icahn Laboratory, Princeton University, Princeton, NJ 08544, USA; 3 Department of Mathematics, Princeton University, Washington Road, Princeton, NJ 08540, USA; 4 Department of Genetics, School of Medicine, Mailstop-S120, Stanford University, Stanford, CA 94305-5120, USA

## Abstract

We have developed a general probabilistic system for query-based discovery of pathway-specific networks through integration of diverse genome-wide data. This framework was validated by accurately recovering known networks for 31 biological processes in *Saccharomyces cerevisiae *and experimentally verifying predictions for the process of chromosomal segregation. Our system, bioPIXIE, a public, comprehensive system for integration, analysis, and visualization of biological network predictions for *S. cerevisiae*, is freely accessible over the worldwide web.

## Abstract 2

BioPIXIE is a probabilistic system for query-based discovery of pathway-specific networks through integration of diverse genome-wide data.

## Background

Understanding biological networks on a whole-genome scale is a key challenge in modern systems biology. Broad availability of diverse functional genomic data from protein-protein interaction, gene expression, localization, and regulation studies should enable fast and accurate generation of network models through computational prediction and experimental validation. Reliability of experimental results varies among data sets and technologies, however, and these data generally provide only pair-wise evidence for biological relationships between genes or proteins. Most cellular mechanisms, on the other hand, involve groups of genes or gene products that behave in a coordinated way to perform a specific biological process. We will refer to such groups of functionally related genes as process-specific networks. Although a wide variety of functional genomic data is available, and much has been learned from them, we are far from exploiting the full potential of these data for discovering such process-specific networks. There are several reasons for this: lack of accessibility to data and methods to analyze them, barriers to incorporating expert knowledge in the network discovery process, and noise and heterogeneity in high-throughput gene data.

The first problem is simply the lack of accessibility of both the data and analysis methods. Even when data are publicly available, results are often buried in large files, and computational methods developed to analyze them are often not available in forms that the typical biologist can use. Thus, experimental researchers are unable to identify interesting results from computational studies that are worth verifying. Instead, most biologists are limited to what the authors of such studies deem important or interesting enough to highlight in the written publication. Our ability to effectively utilize genomic data for process-specific network discovery has thus been hampered by the lack of effective interfaces to both the data and the relevant analysis methods.

The second challenge is to allow biology researchers to integrate their biological knowledge in analysis. When biologists inquire about particular biological processes, they bring with them existing knowledge that can and should be used to generate the most sensitive and precise hypotheses possible. Such information is hard to extract automatically, and effectively incorporating biological expert knowledge is of course closely linked to the accessibility challenge noted above. Most previous methods for process-specific network prediction have not allowed biologists to use their previous knowledge in their area of interest to target the analysis process. Biological research demands convenient and accessible systems that leverage existing knowledge to direct and facilitate discovery.

The third challenge in constructing accurate process-specific networks from diverse genomic data lies in the heterogeneity and high noise levels in large-scale data sets. High-throughput data by nature are often noisy and simple combinations of results from different types of experiments (for example, conclusions of genome-scale two-hybrid experiments and microarray studies) are of limited effectiveness because they sacrifice either sensitivity or specificity.

Recent applications of probabilistic data integration to the related but simpler problem of predicting protein function from diverse genomic data have demonstrated that integrated analysis of heterogeneous sources provides a substantial increase in prediction accuracy. Much of the work in function prediction focuses on fusing information from multiple heterogeneous sources for pairs of proteins to make more reliable statements about pair-wise functional relationships. Bayesian networks [1,2] and variations of this approach [3-5] have been applied successfully to construct 'functional linkage maps' whose connecting edges represent probabilistic support for a functional relationship between the adjacent proteins. Protein functions are then inferred through 'guilt by association' with surrounding nodes of known function. Several studies have formalized this 'guilt by association' approach by using Markov Random Field models to propagate known functional annotations through confidence-weighted edges [6-8].

Despite much investigation into heterogeneous data integration for the purpose of function prediction, there have been only limited attempts to use confidence-weighted linkage maps from integrated data to address the more biologically significant problem of how to group functionally related proteins together into process-specific networks. These network-level questions are distinctly different from function prediction problems and require new methodology for general data integration and network discovery. Previous work in identifying groups of genes involved in specific biological pathways from interaction networks has focused on mainly binary interactions, which are prone to false positives and inadequate coverage when only limited types of genomic evidence are used. For instance, two studies [9,10] describe approaches for finding highly connected subgraphs in binary interaction graphs from high-throughput experiments. They found that highly connected groups in these graphs often correspond to protein complexes or biological processes. Another study [11] introduced the notion of modular decomposition of protein-protein interaction networks to make inferences about pathways. While these approaches have demonstrated the promise of using protein-protein interaction networks for recognizing groups of proteins involved in specific processes, they are constrained by their reliance on limited types of interaction data and their use of binary, rather than probabilistic networks. A recent study extended these approaches to a weighted interaction network and used graph clustering analysis to detect coordinated functional modules [12]. A common theme among many of these studies is their unsupervised approach to network detection. Incorporating expert knowledge in the search process, however, can dramatically improve both the specificity and sensitivity of process-specific network discovery from protein-protein interaction data.

To our knowledge, the only existing work that leverages expert knowledge in constructing biological networks or protein complexes from integrated data is a network reliability approach to protein complex recovery [13] and a greedy search algorithm applied to a confidence-weighted protein-protein interaction network [14]. The former was specifically targeted towards protein complexes, while we focus on the more general problem of discovering not just physically interacting sets of proteins, but functional or process-specific networks. The latter algorithm, proposed by Bader [14], leveraged both physical and genetic interaction data with the goal of extracting more general protein networks. Distinctions between Bader's and our approach are that we integrate functional genomic data in a Bayesian framework that allows a probabilistic, rather than heuristic, graph search. This probabilistic search incorporates both direct and indirect protein-protein links while integrating a wider variety of data (for example, microarray expression, co-localization). Furthermore, we are the first to our knowledge to develop an interactive, web-accessible system that both facilitates discovery of novel biological networks and allows exploratory analysis of the underlying genomic data that support these predictions.

To address these challenges to discovering process-specific networks from functional genomic data, we have created a publicly available system called bioPIXIE (biological Process Inference from eXperimental Interaction Evidence). The system allows users to enter a set of proteins and then uses a novel probabilistic graph search algorithm on a protein-protein linkage map derived from diverse genomic data to predict the surrounding process-specific network for the local neighborhood of interest. Most importantly, the system includes a convenient interface for dynamic visualization of the resulting predictions and provides analysis of their functional coherence. We have completed an extensive evaluation of our method against known pathways as well as experimentally verified a subset of predictions made by our system.

## Results

### Evaluation of the method on known biological networks

Our system achieves accurate network prediction by effectively integrating diverse data sets and probabilistically identifying new components of process-specific networks given only one or a few known members. We evaluated the ability of our approach to recover known process-specific networks given initial query sets by using a collection of well-annotated functional groups, including KEGG pathways, sets of biological process GO terms, and MIPS protein complexes. We restricted our evaluation to groups of 15 to 250 total proteins in which at least half of the member proteins had one type of evidence linking them with another member protein. We identified 31 such groups from the set of KEGG pathways, MIPS protein complexes, and GO terms (see Additional data file 2 and supplemental Table S1 in [15]). We evaluated the performance of our method on each group by sampling 100 random query sets consisting of 10 proteins each from the pathway or complex of interest, applying our data integration and search algorithm, and analyzing the returned set of proteins for consistency with the remaining proteins in the group.

**Figure 1:**
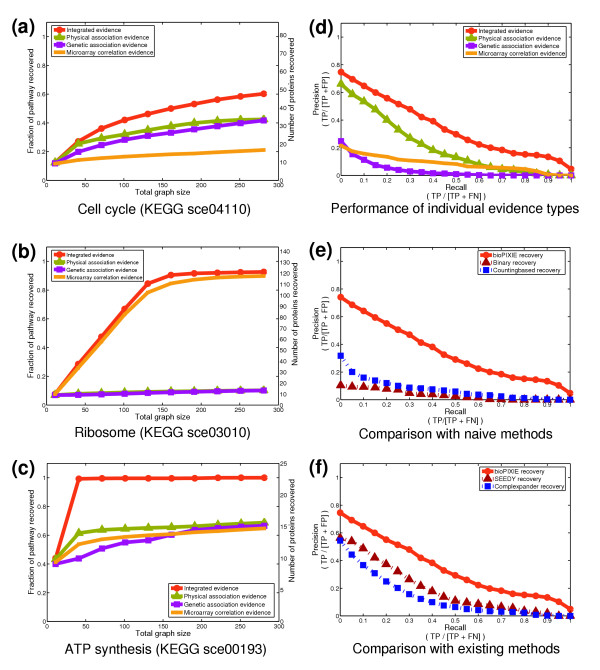
bioPIXIE network recovery evaluation. (**a-c**) Typical network recovery performance for three KEGG pathways. For all pathways, ten proteins from the pathway were randomly picked as a query set. The results of 100 independent query set samplings are shown. The fraction of the total known process components recovered is plotted versus the size of the graph grown from the query set. (**d-f**) An average over 31 KEGG pathways, GO biological processes, and MIPS complexes. Performance is measured and reported as the trade-off between precision (the proportion of correct pathway components returned to the total size of the returned network) and recall (the proportion of correct pathway components returned to the number of total non-query pathway proteins). Precision and recall are derived from true positives (TP), false positives (FP), and false negatives (FN) as noted in the axis labels. (**d**) The improvement gained by using our network prediction algorithm on a Bayesian integration of genomic evidence compared to separate evidence types. bioPIXIE shows considerable improvement in both the number of known member proteins recovered and the precision of predicted members for the integrated evidence over any individual evidence type. (**e**) The improved network recovery offered by the bioPIXIE algorithm versus more naïve approaches to integration and graph search. Specifically, we plot the performance of bioPIXIE on integrated data against a naïve binary approach for which information from all evidence types is used but only as a binary 'yes' or 'no' relationship, and a more sophisticated approach where overlapping evidence receives higher weights and connected proteins are recovered in order of confidence. (**f**) Comparison of the performance of bioPIXIE to two existing methods for query-based protein complex recovery [13,14].

The advantage of using bioPIXIE to integrate multiple types of genomic data is illustrated in Figure 1a-c for three diverse KEGG pathways (graphs for all 31 processes are available in supplemental Figure S2 in [15]). bioPIXIE dramatically and consistently improves the number of network components recovered over any of the individual types of evidence. For example, for KEGG cell cycle proteins (Figure 1a), given a random 10-protein query set, we identified an average of 42 of the remaining 77 proteins using integrated data, whereas only 25 were identified by either physical or genetic evidence, and only 18 by microarray evidence alone. Different evidence types have varying degrees of relevance for different pathways - microarray correlation is very informative for ribosome proteins (Figure 1b) whereas physical interactions are more informative for proteins involved in ATP synthesis (Figure 1c).

This advantage of integrating diverse data types is confirmed in a more comprehensive evaluation of bioPIXIE's performance, where we averaged results over the entire set of 31 processes and complexes described above. Figure 1d compares the precision-recall characteristics of our network identification method using Bayesian integrated data versus using individual evidence types. Given only 10 query genes, the integrated version recovered 50% of the remaining members at a precision of 30% whereas the method applied to independent subsets achieved only 15% (physical association), 10% (genetic association), and 3% (microarray correlation) precision at the same recall (Figure 1d). Thus, combining data from multiple sources clearly improves network recovery.

One might expect that due to the relative sparseness of current functional genomic data, simple combinations of these sources followed by a straightforward search would be sufficient for precise network recovery. However, such combinations are substantially less effective than our approach, as shown in Figure 1e, which plots the average precision-recall characteristics of two such approaches to integration and recovery. The first approach ('Binary recovery') uses all available evidence, but only as a binary 'yes' or 'no', depending on whether evidence of any type is present for a particular protein pair. Given a query, connected proteins are then added in an arbitrary order. The second approach ('Counting-based recovery') also uses all available evidence but counts observed evidence for each pair such that overlaps between multiple sources of evidence receive higher weights. Proteins are then added in order of weight for network recovery. Neither of these simpler approaches achieves accuracy similar to that of our method. In fact, the counting-based approach yields a 4-fold lower prediction precision than our approach and the binary approach results in a 10-fold lower prediction precision at 50% recall.

In addition to these two naive methods, we have also compared our system to two previously published methods for query-based protein complex discovery, SEEDY [13] and Complexpander [14]. bioPIXIE's performance is superior to both existing methods; it achieves an average of 30% precision at 50% recall while SEEDY yields 12% and Complexpander 7% at 50% recall (Figure 1f). Furthermore, calculating the average area under the precision-recall curve (AUC) for each pathway individually, we find that the average bioPIXIE AUC exceeds the average SEEDY AUC by more than one standard deviation for 22 of the 31 groups, while SEEDY outperforms only bioPIXIE for only 1 of the 31 groups (Additional data file 3 and supplemental Figure S4 in [15]). Similarly bioPIXIE outperforms Complexpander for 26 of the 31 groups, while the converse never occurs (Additional data file 3 and supplemental Figure S4 in [15]).

There are several reasons for the superior performance of bioPIXIE. A major factor in its improvement is the robust integration of a wide variety of genomic data. Both Asthana *et al*[13] and Bader [14] focused their integration methodology on physical interactions data (two-hybrid and affinity precipitation data). Our goal is to predict process-specific networks rather than only complexes, which requires a more general integration method applicable beyond physical interactions. These diverse data types have varying degrees of information across different complexes and processes, as evident from the three KEGG pathways illustrated in Figure 1 and a broader study of bioPIXIE's performance on subsets of evidence (see Additional data file 3). Our Bayesian integration can robustly incorporate these data, which allows us to harness the information from heterogeneous data types without sacrificing specificity.

The search algorithm applied to the resulting integrated probabilistic network is also a factor in bioPIXIE's improvement over existing approaches. Our algorithm incorporates information about both direct and indirect links between candidate proteins and the query set in a way that favors tightly connected groups. SEEDY returns the weight of the maximum confidence link between a candidate protein and any member of the query set, which only takes into account direct connections and uses little information about the topology of the network. Furthermore, the maximum is susceptible to noise in both the query set and weights between pairs of proteins. A single erroneous high-confidence link can bring a candidate protein into the result set. The other algorithm included for comparison, Complexpander, samples several random binary networks whose edges are present with probability corresponding to the confidence in that interaction. Proteins are ranked by the fraction of random networks in which there exists a path, up to a maximum length (default of four), from each protein to the query set. Although this algorithm uses more information than SEEDY, both in terms of topology and indirect links, we found its performance to scale poorly with increased density of the weighted interaction network. Specifically, as more genomic data are included in the integration, the probabilistic integrated network becomes more populated, resulting in many more possible (probability >0) paths between any one protein and a particular query set. There are so many paths that the fraction of random binary networks with paths to the query set is no longer a discriminative measure, which results in more false positives. Although such a method might be appropriate for sparse data, it does not appear to work well when larger datasets are applied to the problem of query-based complex or pathway recovery.

Another factor in the performance of our method is its robustness to the quality and size of the query set. For each of the 31 groups of proteins described earlier, we evaluated the recovery performance for 20 query proteins, of which between 1 and 19 were randomly chosen from the entire proteome and the rest were chosen from the appropriate process or complex. All 31 groups could tolerate 25% query set noise with less than a 10% reduction in the average AUC; 27 of those could tolerate 50% query set noise, and 14 of those could tolerate up to 75% random proteins in the query set (see supplemental Figure S5 in [15]). Thus, our method is robust to imperfect query sets. We also evaluated the recovery performance over a range of query set sizes from 4 to 60 proteins to determine whether there was a noticeable decline in performance for very small query sets. We found that, in general, the quality of the network recovered from a pure query set of 4 to 5 proteins is comparable to the result of a much larger query (40 to 50 proteins) on the same process, suggesting that relatively few proteins are required to obtain a signal (supplemental Figure S6 in [15]). For instance, with only a 4-protein query set, bioPIXIE's maximum AUC score was within 10% of the maximum AUC score obtained on up to 60-protein query sets for 22 of the 31 processes (see supplemental Figure S6 in [15] for supporting plot).

**Figure 2:**
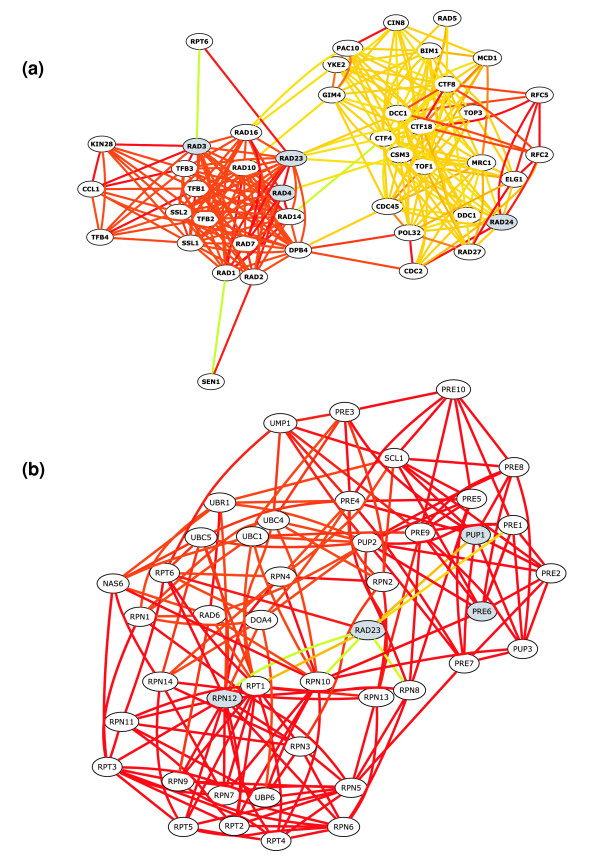
bioPIXIE query-driven context illustration. Nodes represent proteins, and edges represent functional links between them. Edge color indicates the confidence of the links ordered by color from red (highest confidence), orange, yellow, to green (lowest confidence). Query proteins are indicated by gray nodes. Rad23 is known to form a complex with Rad4 (NEF2) and participate in nucleotide excision repair and has also been implicated in inhibiting the degradation of specific substrates in response to DNA damage. (**a**) Rad23 was entered with Rad4, Rad3, and Rad24 and the resulting network is enriched (22 of 44, *P *value < 10^-22^) for DNA repair proteins (GO:0006281). (**b**) Rad23 was entered with proteasome components Pup1, Pre6, Rpn12 and the recovered network is enriched (36 of 44, *P *value < 10^-55^) for ubiquitin-dependent catabolism proteins (GO:0006511) and only contains 2 DNA repair proteins (Rad6 and Rad23). Rad23 has high-confidence relationships with several proteins in both processes, but the network recovery algorithm is dependent on the context of the query, which results in two different views of Rad23 and its neighbors.

The query-driven nature of the search algorithm is a key factor in the accuracy of our method. The relationships between query proteins selected by the user affect which neighboring proteins are added to the final network. Thus, the network resulting from a query is not simply a sub-section of the complete integrated protein-protein interaction graph rooted at the query proteins; rather, it is probabilistically biased by the network search algorithm toward the specific biological context represented in the query set. Figure 2 illustrates this effect for the query protein Rad23. Rad23 is known to form a complex with Rad4 (NEF2) and participate in nucleotide excision repair [16]. Recent work has also suggested that Rad23 facilitates DNA repair by inhibiting the degradation of specific substrates in response to DNA damage [17,18]. Depending on which partners are included in a query with Rad23, the network recovered by our system can focus on Rad23's involvement in nucleotide excision repair or in ubiquitin-dependent protein catabolism. For instance, when the query includes DNA repair proteins Rad4, Rad3, and Rad24 in addition to Rad23, the recovered network of 44 total proteins (Figure 2a) is highly enriched for DNA repair (GO:0006281), with 22 of the 44 having direct or indirect annotations (*P *value < 10^-22^). However, when Rad23 is entered as a query with proteasome components Pup1, Pre6, Rpn12, the resulting network (Figure 2b) is instead enriched for ubiquitin-dependent catabolism (GO:0006511), with 36 of the 44 having direct or indirect annotations (*P *value < 10^-55^). Rad23 has high-confidence relationships with several proteins in both processes, but the recovered network returned by our system is dependent on the context implied by the query. This query-driven context facilitates accurate recovery of network components related to the biological process or pathway of interest.

### Experimental validation of novel network components

**Figure 3:**
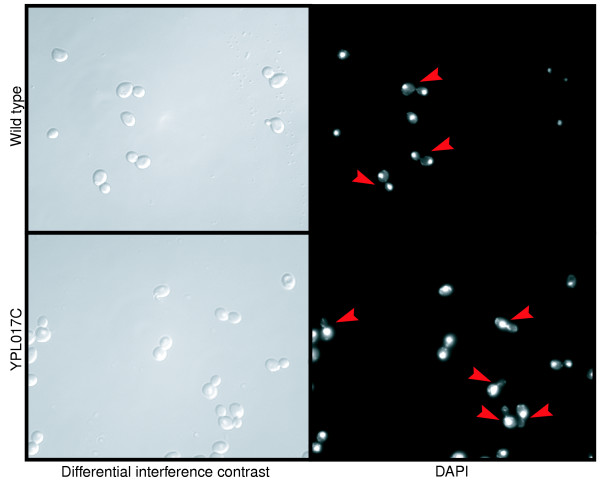
Experimental validation of bioPIXIE prediction for the biological role of YPL017C. bioPIXIE was used to predict previously uncharacterized genes likely to participate in processes related to chromosomal segregation (data for YPL017C shown). Yeast cells were fixed, stained, and photographed using differential interference contrast imaging and 4'-6-diamidino-2-phenylindole (DAPI) staining. When compared with wild-type cells, populations of cells lacking YPL017C have a higher proportion of large-budded cells with a single nucleus at the bud neck (75% compared to 22% in wild type, Fisher exact test *P *value of 5 × 10^-9^). Large budding cells are indicated by arrows. This morphology and failure of nuclear separation are analogous to that of ctf4Δ mutants [19], supporting the hypothesis that YPL017C, like CTF4, is involved in chromosome segregation. See Figure S8 in [15] for experimental verification of YPL077C and YPL144W.

bioPIXIE does not simply recapitulate known biology, but it also predicts novel network components based on the diverse types of input data. In fact, the 'false positives' identified by bioPIXIE in the evaluation above may be novel discoveries or known proteins that interact very closely with the biological process in question but are not annotated to it by the current standard. Thus, although the computational evaluation above is an accurate comparative evaluation of the methods, we wanted to experimentally confirm the quality of predictions made by our method. We have done so by using bioPIXIE to generate hypotheses about previously uncharacterized proteins in yeast and experimentally testing these hypotheses. Specifically, for several biological processes of interest, we entered member proteins as queries and identified uncharacterized proteins consistently returned in the predicted networks. One biological process with high-confidence uncharacterized proteins was the process of chromosomal segregation. In yeast strains null for these genes (YPL017C, YPL077C, and YPL144W), we observed a significantly increased number of large-budded cells with a single nucleus at the bud neck compared to wild-type populations (for example, 75% compared to 22% in wild type, Fisher exact test *P *value of 5 × 10^-9 ^for YPL017C), which is consistent with the phenotype of mutants known to affect chromosome segregation such as ctf4Δ [19] (Figure 3 and supplemental Figure S8 in [15]). This example demonstrates that bioPIXIE facilitates experimental design by providing high-confidence predictions that can be readily tested experimentally using standard molecular biology techniques. Overall, we have observed 1,006 uncharacterized yeast genes with links to known biological processes, and we are able to make high-confidence predictions for 92 of them (supplemental Table S3 in [15]).

### Example use of the system: Prediction of novel targets for the Cdc37-Hsp90 complex

We expect that bioPIXIE will be a convenient and effective tool for biologists to explore the growing sets of functional genomic data as well as direct further experimentation in their domains of interest. As an example of this type of exploratory analysis, we used bioPIXIE to examine the Cdc37-Hsp90 complex and found evidence for previously uncharacterized roles in important processes. Hsp90 is a molecular chaperone that participates in the folding of several proteins, including signaling kinases and hormone receptors, which are involved in growth and apoptotic pathways; it has thus been identified as a possible anticancer drug target. Hsp90 is a highly conserved protein found in organisms from bacteria to humans, and there are two Hsp90 homologs in yeast, HSC82 and HSP82 (reviewed in [20-22]).

**Figure 4:**
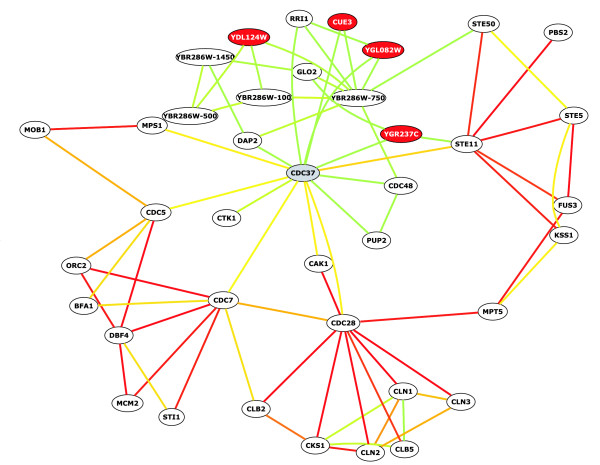
bioPIXIE output for Cdc37. Nodes represent genes, and edges represent functional links between them. Edge color indicates the confidence of the links ordered by color, from red (highest confidence), orange, yellow, to green (lowest confidence). In this example, *CDC37 *was entered as input (gray node); other genes displayed (white nodes) were identified by the bioPIXIE prediction algorithm. Red nodes indicate that the gene is uncharacterized. These results and networks for other proteins can be viewed at [54].

Using bioPIXIE, we were able to identify known and novel targets of Hsp90 and its co-chaperones, in particular Cdc37. Cdc37 and other proteins associated with Hsp90 are thought both to function as chaperones themselves and potentially to determine Hsp90 target specificity. Cdc37 interacts with Hsp90 and is involved in the folding of protein kinases (CDKs, MAP kinases), and previous work has suggested that Cdc37 might be a general kinase chaperone [23]. When Cdc37 is entered as a seed protein into bioPIXIE, our algorithm detects associations between Cdc37 and several kinases that are known interaction partners (Cdc28 [21,24,25], Mps1 [26], Cak1 [24,25], Ste11 [27,28], Cdc5 [24]) (Figure 4). In addition, bioPIXIE predicts previously uncharacterized connections between Cdc37 and the protein kinase Ctk1, based on high-throughput affinity precipitation, thus providing further support for the hypothesis that Cdc37 may be a general kinase chaperone.

Furthermore, our algorithm predicts a potential novel role of the Cdc37-Hsp90 complex in DNA replication. Specifically, bioPIXIE identifies connections between components of this complex and Cdc7, a serine/threonine kinase involved in replication origin firing, which is regulated by Dbf4 in a manner analogous to the way that CDKs are regulated by cyclins [29]. Our system predicts this interaction (confidence of 0.49) based on a combination of two hybrid evidence and correlated expression data. Although this putative interaction was identified in a two hybrid screen, it was not further characterized [24]. In further support of the DNA replication link, bioPIXIE also identifies previously uncharacterized interactionsbetween Cdc7 and two other members of the Hsp90 complex, Sti1 and Cpr7(supplemental Figure S9 in [15]). Sti1 is also functionally linked to Dbf4, a regulator of Cdc7, by the algorithm on the basis of a high-throughput genetic interaction [30] and correlated gene expression in a microarray experiment [31]. Because our system integrates diverse data sources, it highlights interesting interactions that may otherwise go unnoticed. Furthermore, bioPIXIE's network identification and interactive exploration features allow generation of novel, experimentally testable hypotheses, in this case that Cdc37-Hsp90 complexes may have a previously uncharacterized role in some aspect of DNA replication.

### Functional links across biological pathways

**Figure 5:**
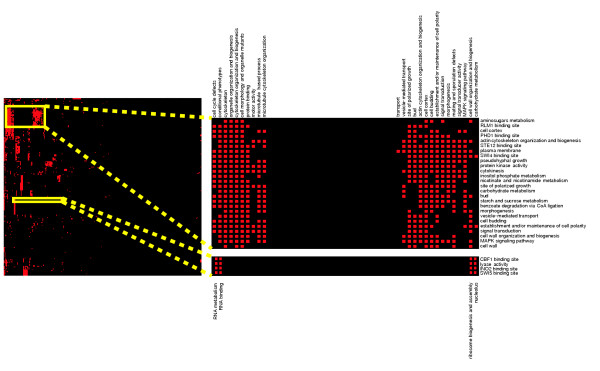
A map of cross-talk between 363 biological groups in *S. cerevisiae*. The combination of our Bayesian data integration system and our network discovery algorithm allows us to find biologically significant cross-talk among known biological groups. The interaction matrix was generated based on 363 KEGG pathways, GO categories, and co-regulated transcription factor targets. Rows of this matrix correspond to the query group and columns correspond to potential cross-talk partner processes; red boxes signify statistically significant links. The cross-talk matrix has been clustered [58] to reveal tightly connected groups of interacting processes (clusters in this matrix correspond to sets of groups who interact with same partners). Highlighted clusters are discussed in the text. See supplemental Figure S10 in [15] for a complete, labeled map.

Our approach of combining data integration with a method for process-specific network discovery provides a convenient framework for addressing biological questions at a higher level. Thus, in addition to constructing specific and testable hypotheses about individual biological processes, we can use the system to discover novel interplay, or cross-talk, among biological networks. To investigate possible cross-talk among biological networks, we start with a single functional group as our query set, use bioPIXIE to predict additional network components, and analyze the resulting superset of proteins for statistical enrichment of other functional groups. By repeating this for each process of interest, we can construct a map of cross-talk that represents a variety of high-level biological relationships (see Materials and methods for details of this analysis). We have applied this approach to map functional links among a set of 363 KEGG pathways, GO categories, and co-regulated transcription factor targets. By using this variety of classification systems, we can detect links across different biological relationships - from biological roles (GO process ontology) to cellular locations (GO component ontology) to metabolic pathways (KEGG). Upon mapping cross-talk among these groups, we clustered the results to reveal biologically significant groups of inter-related processes (Figure 5 and supplemental Figure S10 and Table S4 in [15]).

This analysis identifies several known or expected relationships between networks with related functions. For example, one would expect that the processes of actin cytoskeleton organization, vesicle-mediated transport, and budding would be well connected with each other, and that proteins involved in these processes would share similar functional links to proteins localized to the sites of polarized growth or proteins that when mutated cause morphological defects. Indeed, these groups of genes are found in a tight cluster in our cross-talk analysis (Figure 5, top cluster).

In addition to such clusters that are expected based on current biological knowledge, we also identified novel relationships. For example, one such cluster contains four previously unrelated groups, namely genes that have Swi5 binding sites, genes with Ino2 binding sites, proteins with lyase activity, and genes that have Cbf1 binding sites. Swi5 activates genes expressed at the M/G1 boundary and during G1 phase of the cell cycle, and Ino2 regulates expression of phospholipid biosynthetic genes. Cbf1 is required for the function of centromeres and MET gene promoters, and recent work suggests a general role for Cbf1 in chromatin remodeling [32]. These four groups are found in the same cluster because they share significant links with ribosome biogenesis and assembly, nucleolus, RNA binding, and RNA metabolism. This suggests an explicit, functional link among the processes of cell cycle regulation, transcriptional regulation, inositol metabolism and protein synthesis.

Although the cross-talk across all of these biological processes has not yet been well characterized, evidence in the literature supports these predicted connections.

For instance, the expression pattern of CBF1, INO2, or SWI5 is well correlated with the expression of NOP7 (for example, as cells undergo diauxic shift and during sporulation, CBF1 and NOP7 are co-expressed with a Pearson correlation of greater than 0.8 [33-35]). Du and Stillman [36] found that Nop7/Yph1, a protein required for the biogenesis of 60S ribosomal subunits [37-39], associates with the origin recognition complex, cell cycle-related proteins, and MCM proteins. As cells are depleted of Nop7p, they exhibit cell cycle arrest, and in wild-type cells, Nop7 levels vary in response to different carbon sources [39]. Taken together, these previous experimental results support our prediction linking metabolic pathways, the cell cycle, and ribosome assembly. It is important to note that while the characterization of Nop7 is consistent with this prediction, the individual experiments with Nop7 described above were not part of the input data to our system. Rather, our system was able to make the predicted links across these functional groups based on other heterogeneous, and mostly high throughout, data through bioPIXIE integration and network analysis. Thus, cross-talk analysis using bioPIXIE is effective in identifying novel interplay among pathways, biological processes, cellular locations, and regulatory modules.

## Discussion

We have developed bioPIXIE, an analysis and visualization system for the discovery of biological process-specific networks. bioPIXIE's public interface allows researchers to use their knowledge to explore novel and previously known components of a variety of biological processes. The system provides detailed information about experimental sources for each prediction, including links to original literature, and can be used to generate testable hypotheses. It is important to note that predictions made by bioPIXIE require further experimental validation; we hope that the public availability of our system and all results presented here will encourage such verification by yeast biology laboratories.

A key strength of our system is in addressing network-level behavior as opposed to focusing purely on pair-wise protein relationships. This is critical because many biologically significant questions involve the behavior of groups of proteins in networks or the interplay among networks with different functions. Furthermore, from a computational standpoint, the network-level approach to analysis and modeling of biological data is beneficial because subtle but coordinated group behavior can provide a more accurate picture of biological relationships than can be detected through pair-wise protein linkages. Although we focus on discovering networks, bioPIXIE can also be used for function prediction of individual proteins. Functions of uncharacterized proteins can be predicted either by analyzing uncharacterized components that are returned by the system given a known query set or by using an uncharacterized protein itself as the query, building the local interaction graph around it with our network-discovery algorithm, and analyzing the proteins in the final graph for statistical enrichment for particular functions. Another advantage of bioPIXIE is the probabilistic nature of the method that can easily adapt to new types of data. In the future, bioPIXIE will incorporate additional data sets from sources already modeled by the system as well as data from new approaches such as protein microarrays.

Another future direction for our method is to use process-specific neighborhoods generated by the system as a starting point for deciphering more precise details of biological relationships. Our notion of functional relationship is intentionally rather general so a wide variety of biological interactions can be detected. However, developing detailed models of how groups of functionally related proteins specifically relate to each other requires more precise definitions of relationships. We propose our method as a way to pinpoint groups of proteins acting together, after which other methods can be applied to investigate details of relationships between these proteins. This narrowing process will undoubtedly improve downstream computational approaches.

Finally, our method may be applicable to higher eukaryotes. Additional challenges for such applications include handling multiple cell types, less comprehensive sets of functional genomics data, and incomplete genome annotation. Our method is general, and by extending the Bayesian network structure to organism-specific data sources and learning the corresponding integration weights from available annotation data, bioPIXIE can enable discovery and accurate modeling of previously uncharacterized process-specific networks in a diverse range of organisms. It is important to stress that the success of applying our method and other related approaches to higher eukaryotes depends on public availability of functional genomics data for these organisms and continued improvement of their annotation data, ideally through expert curation.

## Conclusions

We have developed a novel probabilistic methodology for identification of biological process-specific networks based on diverse genomic data and have used this methodology to create a fully functional system for network analysis and visualization. bioPIXIE allows researchers to identify novel pathway components and to study specific interactions among them. Predictions made by our system are specific enough to be tested using common molecular biology techniques. Using this approach, we have accurately modeled multiple known processes in *Saccharomyces cerevisiae*, characterized unknown components in these processes, and identified novel cross-talk relationships. We are making bioPIXIE publicly available through the web to ensure that analysis and interpretation of accurate network predictions we generate, as well as the underlying data, are conveniently accessible to biological researchers.

## Materials and methods

**Figure 6:**
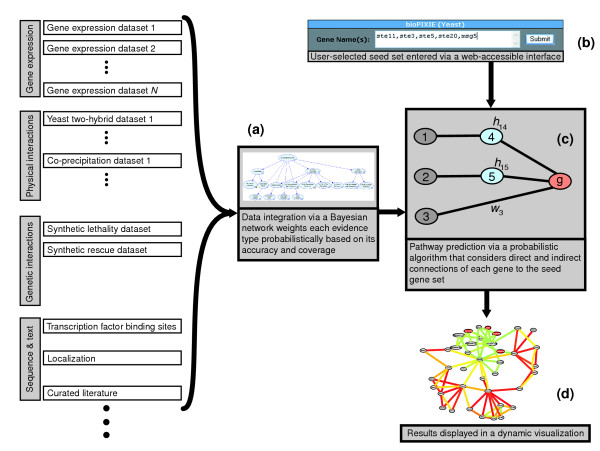
Overview of the bioPIXIE system. Diverse data sets are integrated with a Bayesian network, which weighs each evidence type probabilistically based on its accuracy (**a**). This Bayesian integration produces a graph with confidence-weighted relationships between each gene pair (characterized in supplemental Figure S1 in [15]). Based on this integrated network graph and a user-defined query set of proteins of interest (**b**), the network prediction algorithm identifies novel network components by finding proteins with the maximum expected number of direct and indirect relationships with the query set (**c**). The resulting network is then displayed to the user using a spring model layout, such that the geometric proximity of genes reflects how related they are to each other, and the edge color reflects the confidence of pair-wise connections (**d**). Details of each component are presented in Materials and methods.

Our method relies on four critical components: Bayesian integration of heterogeneous data; an expert-driven search paradigm; a probabilistic graph search algorithm; and an easily accessible interface for interpretation of the results (Figure 6). In simple terms, bioPIXIE integrates different types of data (for example, gene expression, interaction data, high-throughput or single experiments) using a Bayesian framework that is learned from proteins (or genes) that are known to be functionally linked. This Bayesian data integration step reduces the heterogeneous input data to protein pairs with a score indicating the likelihood that they functionally interact, allowing different types of data to be combined with each other. Then, given a protein or group of proteins as a query set (the expert-driven search component), a novel probabilistic algorithm considers the integrated pair-wise relationships to build a local process-specific network around the query proteins.

### Bayesian integration of heterogeneous data

This component uses a Bayesian network to integrate diverse data to derive a probabilistic linkage map among proteins.

#### Functional genomic input data

We have collected a diverse set of evidence from over 950 publications from several databases, including complete physical and genetic interaction data from the GRID and BIND databases (downloaded on 6/25/04), which contain both high-throughput interaction data sets and some interactions from individual experiments curated from the literature [35,40,41]. We also make use of cellular localization data [42], curated sequence data in the form of shared transcription factor binding sites from the *Saccharomyces cerevisiae *Promoter Database (SCPD) [43], and biological complex curated literature from the *Saccharomyces *Genome Database (SGD) [35]. Additionally, we have collected gene expression data from 10 different microarray studies, totaling more than 300 arrays and 29 distinct biological conditions [31,33,34,44-50]. Pearson correlation between genes across each set of related conditions is used as a measure of similarity. Correlation coefficients in each dataset are converted to Z-scores and combined across datasets. References to all sources of genomic data are listed in [51].

#### Bayesian network structure and conditional probabilities

Given these diverse data, we can answer questions about pair-wise protein relationships using a Bayesian network that leverages our previous work [2]. A Bayesian network essentially weights each evidence type according to a measure of confidence in the source of that evidence and then estimates the posterior probability that a relationship exists between two proteins given all observed data [52]. The critical components of such a network are the structure, which determines relationships between evidence nodes, and the conditional probability tables (CPTs), which capture the reliability of each evidence type. The structure of the network used here is expert-based and derived from our previous work [2]. Unlike our previous work, which also relied on experts for estimating the CPTs, here we generalize the framework and automatically learn the CPT for each evidence type using protein-protein relationships inferred by the GO biological process ontology.

Specifically, we obtained gold standard protein-protein relationships for learning the network CPTs by propagating each biological process annotation up to its ancestors and counting the number of unique annotations per GO term. Because the biological specificity of each term roughly corresponds to the number of total annotations, we chose two thresholds to define the set of positive (functionally related) and negative (not functionally related) protein pairs. Protein pairs whose most specific co-annotation occurs in GO terms of 300 total annotations or less were considered positives, while pairs whose most specific co-annotation occurs in GO terms of 1,000 total annotations or more were considered negatives. The resulting set of positive and negative protein pairs can also be downloaded from the online supplement [15].

Given this set of gold standard pairs, we used the expectation-maximization algorithm [53] to compute the CPTs. As expectation-maximization is guaranteed to identify a local, not global, maximum on the likelihood surface, we computed a reasonable starting point for the algorithm based on independent counting of individual evidence sources. We used a discrete Bayesian network, and continuous-valued microarray expression correlation was discretized into 16 bins (see Additional data file 1 for details). Both the structure and final learned conditional probabilities are available as Additional data file 1 and can also be downloaded as supplemental Figure S1 from [15]. The final probabilistic output of the Bayesian network for the whole yeast proteome can be downloaded from the online supplement in [15]. We have performed cross-validation analysis by excluding all related GO relationships from the gold standard for each pathway we attempt to predict.

### Expert-driven search paradigm

A critical aspect of our method is that we make use of existing expert biological knowledge to improve the accuracy of process-specific network prediction by allowing the biologist to drive the search process. Specifically, the user enters a list of proteins (of arbitrary size) he or she either expects to play a role in the same biological process, or wants to test for functional relationships. Our system then queries the surrounding confidence-weighted network derived from integrated data for additional related proteins. The resulting process-specific network is not a simple sub-section of the complete integrated protein-protein interaction graph; rather it is probabilistically biased by the graph search algorithm (described in detail below) toward the biological process represented in the set of query proteins. This paradigm is based on two important observations: first, detailed knowledge of specific biological processes is typically learned in a directed fashion, not by taking a completely unsupervised view of high-throughput data; and second, novel process-specific proteins can be predicted more precisely when we consider their relationship to groups of known proteins simultaneously. This query-driven process results in a view of the integrated genomic data in the context of the specific process being interrogated. Figure 2, discussed in detail in Results, illustrates this behavior for Rad23, a DNA repair protein.

### Probabilistic graph search algorithm

Given an initial set of query proteins defined by the user, we wish to find other proteins with significant connectivity back to the starting group. It is unrealistic to expect related proteins to have direct connections to all other proteins in the same biological process due to incomplete data. Thus, we measure connectivity back to the original query set via both direct and indirect relationships. A brief overview of the algorithm follows: Starting with a user-defined query set of related proteins, first, find the *n*_1 _direct neighbors with largest connections to the query set. Secondly, find the *n*_2 _direct or indirect neighbors with largest connections to the query set, requiring that all indirect paths pass through proteins from step 1. Finally, return *n*_1 _+ *n*_1 _proteins and associated links.

Because we used a Bayesian approach to data integration, weights of edges connecting pairs of proteins are precisely the posterior probability of a functional relationship between the proteins given all observed evidence for the pair, for example, for each edge weight, *e*_*ij*_, in the integrated network:

*e*_*ij *_= *P *(protein i is functionally related to protein *j *| evidence).

Given this formulation, the existence of any pairwise biological relationship can be treated as a Bernoulli random variable, *X*_*ij*_, with probability of success *e*_*ij*_. The number of direct relationships protein *p*_*i *_shares with the original query set, *Q*, can then be found by summing over all *p*_*i*_'s connections to proteins in *Q*. Letting the random variable *S*_*Q*_(*p*_*i*_) denote this sum, we obtain:



Then, the expected number of direct relationships to the query set for protein *p*_*i *_is:



As not all proteins involved in a particular process will have high-probability direct relationships with other members of the same process, we also need to measure indirect connectivity to the query set. However, from a biological standpoint, not all indirect connections are actually meaningful. We expect there are a limited number of high-probability adjacent neighbors of the query set through which indirect connections are meaningful. Thus, our approach relies on a two-step search approach where a pre-defined number of direct neighbors are found (first neighborhood, referred to as *N*_1_) after which the maximally connected indirect neighbors adjacent to the first neighborhood and the original query set are added (second neighborhood, referred to as *N*_2_). Letting the random variable  denote the number of two-step indirect connections between protein *p*_*i *_and the query set (*Q*) through first neighborhood proteins (*N*_1_), we obtain:



and the expected number of indirect connections through the first neighborhood is:



Here, we implicitly assume independence of X_ij _and X_jk_. This requires that the existence of a relationship between any proteins *p*_*i *_and *p*_*j *_be independent of the relationship between proteins *p*_*j *_and *p*_*k*_, which is a reasonable assumption. Also, we do not consider indirect connections beyond two steps from the query set. We have empirically evaluated the algorithm for more distant indirect relationships, but found the performance on two-step relationships superior. The search algorithm is summarized as follows: Given a user-defined query set, Q, first find



Secondly, find



Finally, return {*N*_1_*, N*_2_}.

We have empirically determined that a first neighborhood of between 10 and 20 proteins (that is, 10 ≤ *n*_1 _≤ 20) provides the best precision and recall over a wide range of biological processes. This was determined by optimizing the difference of recall and impurity (1-precision) with respect to the first neighborhood size. Representative examples and further details are included in supplemental figure S7 in [15]. The number of second neighborhood proteins returned (*n*_2_) reflects a tradeoff between precision and recall as demonstrated in Figure 1. We choose *n*_2 _based on the density of the local network and the limits of the user interface (a typical user is unable to draw useful information from interaction graphs of more than 40 proteins). Thus, second neighborhood proteins are added to the graph until the total number of proteins reaches 40 or no neighbors with links exceeding the prior probability of interaction remain.

### Publicly available interface

We provide public, web-based access to our integrated process-specific network analysis and visualization system [54]. This allows biologists to browse the integrated set of functional genomic data for proteins of interest, and explore our network predictions. Furthermore, users can directly query specific links leading to the reported predictions, an important part of the analysis pipeline.

### Cross-talk analysis method

To measure cross-talk between processes, we start with a single pathway as our query set, build the graph of interactions around this query using bioPIXIE, and analyze the resulting superset of proteins for statistical enrichment of other processes. More specifically, we first remove the original query set from the recovered set of proteins and obtain counts of proteins in the remaining set for every other possible interacting pathway. We then use a hypergeometric test to estimate the significance of the observed counts. For example, suppose we use a query pathway, *Q*, and with a graph of size *X *recover *m *proteins annotated to a different pathway, *R*, of total size *M*. If there are *N *total known proteins in the organism of interest, the probability of observing a number this large or greater under the null assumption that the two pathways do not interact is:



We repeated this calculation for all pairwise combinations of pathways (see list in supplemental Table S2 in [15]). We conservatively corrected for multiple hypothesis testing by Bonferroni correction and only report results with corrected *P *values of < 10^-2^.

### Implementation

The Bayesian network used in integrating genomic data was implemented using SMILE, a C++ library developed by the Decision Systems Laboratory at the University of Pittsburgh [55]. The user interface tool, GeNIe, useful for developing and analyzing Bayesian models, was also used extensively during the development of bioPIXIE [55]. bioPIXIE's web interface is implemented in PHP and all genomic data are stored in a MySQL database. The graph server that performs probabilistic searches and renders results is implemented in C++ and renders graphs in SVG, which allows for user-friendly browsing and interactivity. AT&T's Graphviz [56] is used for layout of all graphs.

## Additional data files

The following additional data are available. Additional data file 1 is a DSL file of the bioPIXIE Bayesian network for genomic data integration. This file contains the structure and final learned conditional probability tables used for integrating multiple heterogeneous sources of functional genomic data. GeNIe, available at [57], is recommended for viewing the DSL file. Additional data file 2 contains a list of pathways and protein complexes that were used to evaluate the performance of bioPIXIE. The source of the group and the number of proteins in each is also included. Additional data file 3 contains a comparison of the performance of bioPIXIE to existing methods for biological network recovery.

## Supplementary Material

Additional data file 1 This file contains the structure and final learned conditional probability tables used for integrating multiple heterogeneous sources of functional genomic data. GeNIe, available at , is recommended for viewing the dsl file. Click here for file

Additional data file 2 This file contains a list of pathways and protein complexes that were used to evaluate the performance of bioPIXIE. The source of the group and the number of proteins in each is also included. Click here for file

Additional data file 3 This file contains a comparison of the performance of bioPIXIE to existing methods for biological network recovery. The area under the precision-recall curve (AUC) is computed and plotted separately for each of the 31 evaluation pathways and complexes. Click here for file
